# Hemoglobin Variants in Patients With Microcytic Hypochromic Anemia: A Review of Indian Studies

**DOI:** 10.7759/cureus.38357

**Published:** 2023-04-30

**Authors:** Kaustubh Kharche, Arvind Bhake

**Affiliations:** 1 Department of Pathology, Jawaharlal Nehru Medical College, Datta Meghe Institute of Higher Education and Research, Wardha, IND

**Keywords:** hemoglobinopathies, thalassemia, high performance liquid chromatography (hplc), hemoglobin variants, microcytic hypochromic anemia

## Abstract

Microcytic hypochromic (MCHC) anemia with hemolytic components is common in clinical practice. Hemoglobinopathies and variants are one of the important underlying causes of MCHC anemia. The Indian population, by large, as various studies reported, showed a plethora of hemoglobinopathies with regional predilections for its types. The present systematic review is carried out for the evaluation of MCHC anemia for its underlying causes of hemoglobinopathies and their loco regional comparisons. The review was carried out by the Preferred Reporting Items for Systematic Reviews and Meta-Analyses (PRISMA) method with selected keywords through the Google advanced search matchable to the objectives of the present systematic review. Upon the systematic review, it was observed that β thalassemia trait (βTT) remained the highest reported hemoglobinopathy. The other abnormal hemoglobin variants, though rare, also have been reported in the reviewed articles. It is concluded that patients with MCHC refractory to its regular treatment should be subjected to high-performance liquid chromatography (HPLC) in exclusion of underlying hemoglobinopathy and abnormal hemoglobin variants.

## Introduction and background

Hemoglobinopathy presents as microcytic hypochromic (MCHC) anemia in clinical practice. Hemolytic anemia usually accompanies underlying causes of various hemoglobinopathies. Quantitative and qualitative hemoglobin disorders are one of India's major health concerns. It, therefore, forms a major public health problem [1]. Hemoglobinopathies, whether major or minor, have a genetic basis and are mostly inherited diseases. The governments must allocate the finances to diagnose and treat patients with hemoglobinopathies in a major way to reduce morbidity and mortality. Prenatal diagnosis of hemoglobinopathies and counseling to avoid these hemoglobinopathies are in practice to some extent. The spectrum of hemoglobinopathies in India is wide [1-2]. The studies published in India have documented that thalassemia major and minor form the major volume of sufferers of hemoglobinopathies [1-3]. Minor hemoglobinopathies such as Hb Q India and Hb Lepore too are reported significantly in the Indian population. Hb S of homozygous and heterozygous types as well as Hb S with β thalassemia have also been reported in large numbers in a few studies in India [4-5].

The reports from various parts of India are consistent with the observation that β thalassemia is a major hemoglobinopathy. High-performance liquid chromatography (HPLC) enabled the detection of minor hemoglobinopathy largely as could be gauzed by published reports [6-8].

The comparative reviews on hemoglobinopathies and hemoglobin variants in the context of the Indian subcontinent are not so frequent [9-11]. Therefore, the authors of this study decided to bring about a systematic review of hemoglobinopathies and hemoglobin variants reported from India [12-14]. The present review aims to compare the types and frequency of various hemoglobin variants as gathered from Indian studies published between 2010 and 2021 with anemia (MCHC). The inclusion of the studies for the present review was based on the defined objective above and the uniformity of the methodology adopted.

## Review

Material and methods

Search Strategy

**Figure 1:**
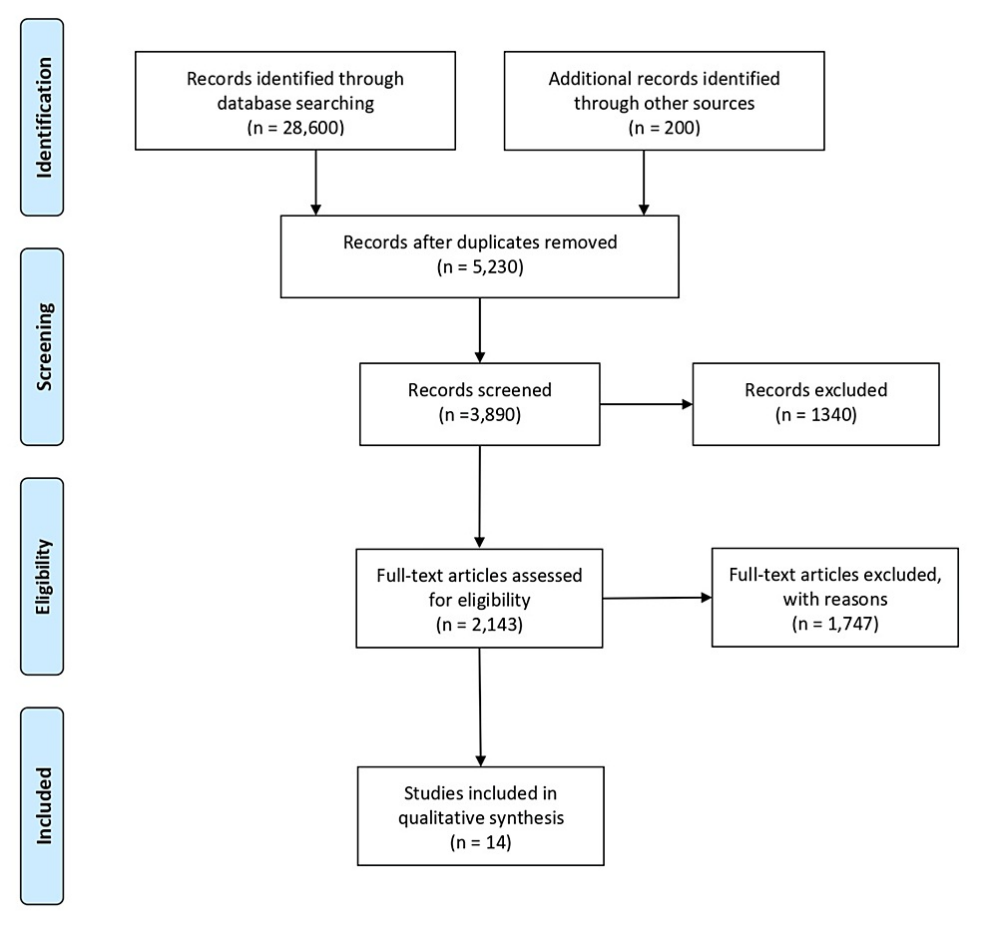
Flow diagram of the literature review (PRISMA). PRISMA, Preferred Reporting Items for Systematic Reviews and Meta-Analyses

The search strategy was defined by the year of publication, the keywords, objectives, and methods in the advanced Google search engine by Preferred Reporting Items for Systematic Reviews and Meta-Analyses (PRISMA). The duration was from 2010 to 2021. The keywords selected for the search were microcytic hypochromic anemia, abnormal hemoglobin variants, HPLC, and India (Figure 1).

Hematological Investigations

The hematological investigations that were taken into account for this study included complete blood counts, hematocrit, mean corpuscular volume (MCV), mean corpuscular hemoglobin (MCH), MCH concentration (MCHC), and red cell distribution width (RDW). These parameters were taken into consideration for the selection of studies to be included in the present review with the understanding that the β thalassemia trait (βTT) and hemoglobin variants morphologically and volumetrically are diagnosed as microcytic hypochromic anemia with coexisting features of mild to marked hemolysis. All the 14 articles selected for the review contained this information (Table 1).

**Table 1:** Hemoglobin variants with types and frequencies. *n*, total number of cases detected; Hb, hemoglobin; βTT, β thalassemia trait; βTM/I, β thalassemia major/intermedia; HPFH, hereditary persistence of fetal hemoglobin; Thal, thalassemia

Sr. no.	Authors, year of publication, no. of cases (n)	Hemoglobin variants										
		βTT	βTM/I	Hb D Punjab	Hb E	Hb Q India	Hb S	Hb J Meerut	Hb D Iran	Hb Lepore	HPFH	Thal + Variant
1.	Sachdev et al. [1], Jan. 2010 (327)	232 (8.9)	56	13	7	5	3	1	1	1	-	8
2.	Chandrashekhar and Soni [2], May 2011 (543)	206 (37.9)	14 (3.1)	5 (0.8)	229 (42.1)	-	29 (6.7)	1 (0.1)	-	1 (0.1)	-	52 (9.4)
3.	Shrivastav et al. [3], Sept. 2013 (1,615)	839 (11.5)	308 (4.2)	58 (0.8)	21 (0.2)	4 (0.06)	299 (4.1)	-	-	2 (0.03)	8 (0.11)	13
4.	Baruah et al. [4], June 2014 (5,320)	313 (3.4)	32 (0.3)	-	4186 (46.5)	-	392 (4.3)	8 (0.09)	-	-	5 (0.09)	200 (2.5)
5.	Iyer et al. [5], Feb. 2015 (8,029)	1821 (22.6)	116 (1.37)	222 (2.7)	262 (3.2)	72 (0.9)	3606 (44.9)	1 (0.01)	5 (0.06)	1 (0.01)	4 (0.05)	290 (3.6)
6.	Bhalodia et al. [6], March 2015 (43)	26 (5.2)	4 (0.8)	2 (0.4)	1 (0.2)	-	6 (1.2)	-	1 (0.2)	-	1 (0.2)	2 (0.4)
7.	Alam et al. [7], July 2015 (226)	62 (18.7)	11 (3.3)	4 (1.2)	8 (2.42)	-	34 (25.6)	-	-	-	-	52 (15.7)
8.	Biswas and Philip [8], March 2016 (740)	522 (8.03)	42 (0.65)	12 (0.18)	67 (1.03)	2 (0.03)	85 (1.3)	-	-	-	1 (0.02)	9 (0.12)
9.	Raman et al. [9], Feb. 2017 (293)	48 (6.1)	8 (1.01)	1 (0.1)	7 (0.8)	-	197 (25.0)	-	-	1 (0.12)	-	27 (3.4)
10.	Sarvaiya and Chauhan [10], May 2017, (386)	216 (10.6)	21 (1.02)	6 (0.29)	1 (0.04)	-	115 (5.6)	-	-	-	7 (0.3)	17 (0.8)
11.	Warghade et al. [11], Aug. 2017 (1,2131)	7,377 (11.2)	529 (0.8)	379 (0.5)	742 (1.1)	50 (0.08)	2,373 (3.6)	46 (0.07)	-	-	98 (0.1)	-
12.	Shankar et al. [12], July 2019 (120)	23 (11.5)	14 (7.0)	2 (1.0)	1 (0.5)	-	43 (21.5)	-	-	-	-	37 (18.5)
13.	Jain and Saxena [13], Oct. 2019 (1,236)	208 (16.8)	3 (0.2)	2 (0.1)	1 (0.08)	-	933 (75.4)	-	-	-	-	-
14.	Ankur et al. [14], Aug. 2021 (858)	586 (21.0)	149 (5.3)	13 (4.04)	24 (0.8)	-	27 (1.0)	-	-	6 (0.2)	15 (0.5)	34 (1.31)
	Total (*n*): 29,293 cases	12,479	1,307	719	5,557	133	8,142	57	7	11	139	741
	Frequency/Percentage	42.6%	4.41%	2.4%	18.9%	0.45%	27.7%	0.19%	0.02%	0.03%	0.47%	2.5%
	This study (*n* = 68)	10 (7.51)	20 (15.03)	-	-	-	20 (15.03)	13 (9.77)	-	-	-	-

Selection Criteria for the Studies

The study selection criteria are mentioned in Table 2.

**Table 2:** Selection criteria for the studies. HPLC, high-performance liquid chromatography

Inclusion criteria	
1	Studies carried out on HPLC for detection of abnormal hemoglobin variants from India
2	The minimum population investigated in the study for abnormal hemoglobin variants more than 200 subjects
3	The studies that were published in Indian literature between the years of 2010 to 2021
4	The studies deemed to be included only from the journals that were indexed
Exclusion criteria	
5	The studies that did not match to the specific objectives of the present review
6	The studies that were not available as online free full text
7	Studies that were available only in the form of online free abstracts

Statistical Methods

The objective of the study was to know the frequency/percentage of abnormal hemoglobin variants in the suspected population and their types. Therefore, the statistical methods used for the comparison of data in the 14 studies were mostly expressed as frequency/percentage.

Results

A total of 14 studies published from different parts of India were selected for review in the duration of 2010 to 2021. Table 1 shows the geographical distribution of publications of articles from India.

The maximum number of studies were published in western and northern India. The total population that was covered within the 14 studies was 29,293. The population that underwent HPLC in 14 studies in the evaluation of MCHC anemia belonged to pediatric as well as adult age groups. The pediatric age group revealed maximum cases of hemoglobin variants. The thalassemia trait was diagnosed mostly in the early years of life as mentioned in these studies.

The abnormal hemoglobin variants reported within the 14 studies and from this study for type and frequency are shown in Table 2. The commonest abnormal hemoglobin variant in all the studies was βTT and with a cumulative percentage of 42.6% [1-14]. The last reported variant in the Indian population was Hb D Iran, with a percentage of 0.02% [1,5,6].

This data is indicative that the rarest hemoglobin variants, as reported in world literature too, are recorded in the Indian population. Furthermore, these little-known hemoglobin variants that cause subclinical or apparent clinical disease remain unsuspected until their hemochromatogram was performed.

The frequency of the major abnormal hemoglobins as could be seen is almost equally distributed in India irrespective of its geographical region for thalassemia was 49.51% and sickle cell disease was 27.79%.

Discussion

The pediatric population dominated at detection of abnormal hemoglobins and hemoglobin variants in the studies reviewed. The total population of 29,293 patients with abnormal hemoglobins and hemoglobin variants in a total of 14 articles showed that βTT was found to be the commonest hemoglobinopathy across all four regions of India. Hb S heterozygous and homozygous constitutes about 27.7% next common hemoglobinopathy across all regions of India in a total of 29,293 patients. Similarly, the Hb S detection was the second commonest detection done on HPLC in all the 14 studies, which were reviewed for this study.

The third most commonest hemoglobinopathy that was detected in the total population of 29,293 patients within the 14 studies was of Hb E. Hb E constituted about 18.9% of hemoglobinopathies. Except for the reports of Sachdev et al. [1], Shrivastav et al. [3], Bhalodia et al. [6], Sarvaiya and Chauhan [10], Shankar et al. [12], and Jain and Saxena [13], Hb E remained the third most common hemoglobin variant detected.

The β thalassemia major (βTM) and Hb D by frequency were the fourth and fifth common hemoglobin disorders in a total population of 29,293 patients. However, their distribution across the region of India was varying. The studies of Shrivastav et al. [1], Iyer et al. [5], Warghade et al. [11], and Ankur et al. [14] recorded higher frequencies of βTM as compared to the other studies reviewed.

Apart from these hemoglobins, other hemoglobin variants too were reported but infrequently in India. Sachdev et al. [1] reported Hb Q India, Hb J Meerut, Hb D Iran, Hb Lepore, and thalassemia variants in a total of 327 patients. Chandrashekhar and Soni [2] reported one case each of Hb J Meerut and Hb Lepore. Shrivastav et al. [3] reported four cases of Hb Q India, two cases of Hb Lepore, eight cases of hereditary persistence of fetal hemoglobin (HPFH), and thalassemia variants in a total of 1,615 patients. Baruah et al. [4] reported eight cases of Hb J Meerut and five cases of HPFH in their study of 5,320 patients. Iyer et al. [5], in their study of 8,029 patients, reported 72 cases of Hb Q India, five cases of Hb D Iran, four cases of HPFH, and one case of Hb J Meerut and Hb Lepore, respectively. Similarly, Bhalodia et al. [6] also reported a single case each of HbD Iran and HPFH.

Biswas and Philip [8], in their study of 740 patients, reported two cases of Hb Q India and a single case of HPFH. Raman et al. [9] reported a single case of Hb Lepore, and Sarvaiya and Chauhan [10] reported seven cases of HPFH. Warghade et al. [11] reported 98 cases of HPFH, 50 cases of Hb Q India, and 46 cases of Hb J Meerut in their study of 12,131 patients. Ankur et al. [14] reported 15 cases of HPFH and six cases of Hb D Iran in their study.

However, there are studies where some of the hemoglobins and hemoglobin variants have not been reported from certain regions of India. The study by Alam et al. [7], Shankar et al. [12], and Jain et al. [13] did not report the abnormal hemoglobins of Hb Q India, Hb J Meerut, Hb D Iran, Hb Lepore, and HPFH, respectively. Similarly, the studies by Alam et al. [7], Raman et al. [9], and Sarvaiya and Chauhan [10] have not reported the presence of Hb Q India, Hb J Meerut, and Hb D Iran.

In this study, β thalassemia major/intermedia (βTM/I) and Hb S were detected with 15.03%. The Hb J Meerut variant was also detected with a percentage of 9.77%. βTT constituted 7.51%. Unlike other studies, this study also detected the Hb C variant with a percentage of 3.75%.

It is evident from all the 14 studies that a sizeable number of the population that presents with anemia, especially the microcytic hypochromic type, have the underlying cause of abnormal hemoglobin and hemoglobin variants. Some other similar studies have also conducted similar research concluding many abnormal hemoglobin variants, but comparison with those studies would be out of the scope of this study [15-20]. The Indian population, which is multiethnic, shows the spectrum of common as well as uncommon hemoglobins and variants in isolation as well as combined hemoglobinopathy irrespective of the geographic regions of India (Table 3).

**Table 3:** Geographical distribution of publication of articles from India.

Sr.no	Geographical area	Studies
1.	Western India	Shrivastav et al. [3], Bhalodia et al. [6], Biswas et al. [8], Jain et al. [13]
2.	Northern India	Sachdev et al. [1], Sarvaiya and Chauhan [10], Shankar et al. [12], Ankur et al. [14]
3.	Eastern India	Baruah et al. [4], Alam et al. [7], Raman et al. [9]
4.	Southern India	Chandrashekhar and Soni [2]
5.	Pan India	Iyer et al. [5], Warghade et al. [11]

## Conclusions

The systematic review carried out over 14 studies across four regions and Pan India surveillance showed that the microcytic hypochromic anemia with hemolytic component refractory to the treatment must undergo the analysis for hemoglobinopathies. The HPLC is probably the best analyzer for detecting the rare hemoglobin variants, which failed to be detected by the electrophoresis. This review further concludes that the anemia in a pediatric population can be due to common hemoglobinopathies such as βTM as well as highly uncommon hemoglobinopathies such as Hb J. Therefore, it is recommended that all pediatric refractory anemia should be evaluated for hemoglobin disorders in the form of traits and diseases.
